# A Difficult Case of Calcineurin Inhibitor Neurotoxicity Post-Haploidentical HCT With a Successful Novel Solution: Cytotoxic T-Lymphocyte-Associated Protein 4-Immunoglobulin Blockade for GVHD Prophylaxis

**DOI:** 10.1177/09636897241265249

**Published:** 2024-07-30

**Authors:** Kaitlyn Dykes, Dimitrios Tzachanis, Divya Koura

**Affiliations:** 1 Division of Hematology Oncology, Moores Cancer Center, University of California San Diego, La Jolla, CA, USA; 2 Division of Blood & Bone Marrow Transplantation, Moores Cancer Center, University of California San Diego, La Jolla, CA, USA

**Keywords:** cancer, graft versus host disease, T cells, bone marrow, oncology

## Abstract

Post–allogeneic hematopoietic cell transplant (HCT) immunosuppression regimens are given as graft-versus-host disease (GVHD) prophylaxis. Most GVHD prophylaxis regimens are based on calcineurin inhibitors (CNIs). Unfortunately, CNIs are associated with significant associated morbidity, frequently cannot be tolerated, and often need to be discontinued. There is no consensus as to which alternative immunosuppression should be used in cases where CNIs have to be permanently discontinued. Cytotoxic T-lymphocyte-associated protein 4-immunoglobulin (CTLA4-Ig) blocking agents are well tolerated and have been used extensively in patients with autoimmune disease and as post-transplant immunosuppression. There are two CTLA4-Ig agents: belatacept and abatacept. Belatacept is routinely used in adult kidney transplantation to prevent rejection and abatacept has been approved by the Food and Drug Administration (FDA) for GVHD prophylaxis in patients undergoing a matched or one allele-mismatched unrelated allogenic HCT. Herein, we describe a case in which abatacept was given off-label to replace tacrolimus GVHD prophylaxis in a patient with neurotoxicity undergoing haploidentical HCT. This case suggests that CTLA4-Ig blockade may be a good alternative to a CNI in cases where the CNI needs to be discontinued and warrants further investigation.

## Introduction

Advances in allogeneic hematopoietic cell transplant (HCT), notably graft-versus-host disease (GVHD) prophylaxis, have led to improved safety of allogeneic HCT over the decades since the first HCTs in the 1980s, thus making allogeneic HCT a possibility for more patients who now undergo allogenic HCT with variable degree of immunologic match^1^. GVHD is a multisystem disorder that occurs when the graft recognizes the host as foreign and attacks the recipient’s body cells through complex T-lymphocyte and cytokine-mediated mechanisms. Acute GVHD (aGVHD) presents generally early in the post-transplant course and is estimated to occur in up to 50% of patients receiving allogenic HCT from a human leukocyte antigen (HLA)–matched sibling. Meanwhile, chronic GVHD (cGVHD) typically occurs later in the transplant course and is reported to occur in 40% of cases, leading to death in 10% of allogenic HCT recipients. Many factors pertaining to the recipient, donor, and type of transplant have been linked to GVHD, with common risk factors including higher degrees of HLA mismatch, older age of recipient or donor, peripheral stem cell recipients, alloimmunization of the donor, and cytomegalovirus (CMV) status^1,2^.

Many GVHD prophylaxis regimens have been published. Generally, prophylaxis includes a calcineurin inhibitor (CNI), such as tacrolimus or cyclosporine combined with an antimetabolite, such as methotrexate or mycophenolate mofetil (MMF)^3^. Additional T-lymphocyte depleting agents can be added to transplant regimens. Antithymocyte globulin (ATG) depletes cytotoxic T-cells and has been shown to both reduce cGVHD and enable more patients to discontinue immunosuppressive medication^4^. Post-transplant cyclophosphamide (PTCy) is also a form of T-cell depletion, thought to ultimately promote Treg-mediated tolerance^5,6^. The GVHD prophylaxis regimen selected is generally guided by predicted risk of GVHD, including type of transplant, recipient and donor features, patient-specific factors that may prohibit certain prophylaxis regimens, and institutional preferences. Specifically, in haploidentical allogenic HCT, current guidelines recommend GVHD prophylaxis with PTCy, CNI, and MMF. Studies have further shown that addition of ATG to non-myeloablative haploidentical HCT is potentially beneficial, with 40% of patients experiencing aGVHD and low occurrence of cGVHD^7^. Despite these improvements, there is a great need for more effective GVHD prophylactic regimens with less associated toxicities. Here, we present a case of a patient who experienced significant CNI toxicity, with a novel solution, cytotoxic T-lymphocyte-associated protein 4-immunoglobulin (CTLA4-Ig) blockade as GVHD prophylaxis^8^.

## Case Presentation

**Figure 1.**
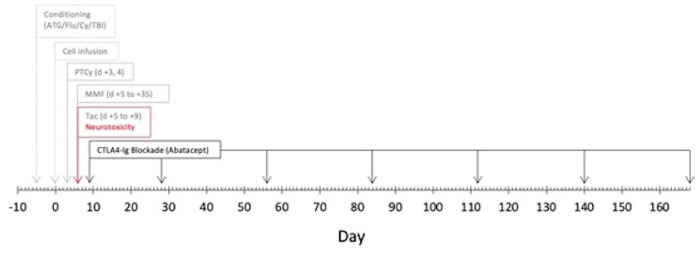
Peri-transplant timeline of calcineurin inhibitor neurotoxicity and graft-versus-host disease prophylaxis regimen. ATG: Antithymocyte globulin; Flu: fludarabine; Cy: cyclophosphamide; TBI: total body irradiation; PTCy: post-transplant cyclophosphamide; tac: tacrolimus: and CTLA4-Ig: cytotoxic T-lymphocyte-associated protein 4 Ig.

**Figure 2.**
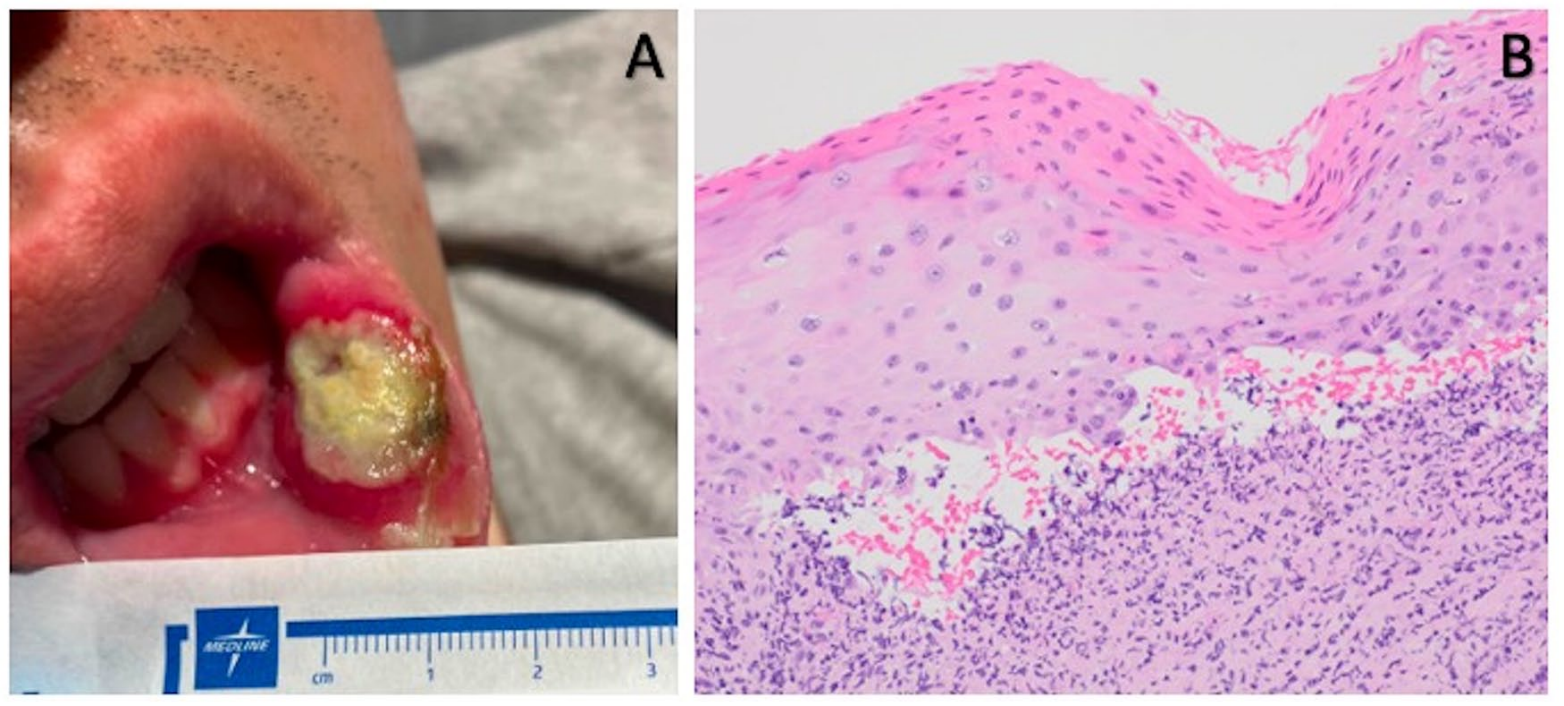
Chronic graft-versus-host disease (cGVHD) oropharynx lesion. Presenting buccal ulceration (A) and high-power view of the tongue biopsy with ulcerated squamous mucosa and scattered dyskeratotic cells with mixed acute and chronic inflammation, which raised the possibility of cGVHD (B).

A 24-year-old man with a medical history of hypoplastic myelodysplastic syndrome (MDS) underwent haploidentical HCT. The donor was the patient’s father. Both the patient and donor were blood type O^+^ and CMV positive. Per the Hopkin’s protocol, he received non-myeloablative ATG, fludarabine (Flu), cyclophosphamide (Cy), and total body irradiation (TBI; Figure 1)^9^. For GVHD prophylaxis, he received PTCy and, on day +5, was started on MMF and tacrolimus continuous infusion of 1.5 mg IV every 24 h. During the hospitalization, he developed significant chemotherapy-related nausea and vomiting, thus received ondansetron, prochlorperazine, and olanzapine scheduled. On day +5, less than 24 h after initiating tacrolimus, the patient developed acute onset diffuse rigidity and jaw dystonia, such that he was unable to extend his tongue, turn his head, and struggled to bend his extremities. Vital signs, other than mildly elevated blood pressure, were stable and within normal. He had no headache or vision changes and neurologic exam was otherwise without deficits. A stroke code was called. Intravenous tacrolimus was paused, and the patient was given diphenhydramine with some improvement in rigidity. Antiemetics prochlorperazine and olanzapine were discontinued. A complete metabolic panel was within normal limits, complete blood counts showed stable treatment and disease-related pancytopenia, and a random tacrolimus level was 6.1 ng/ml. Brain magnetic resonance imaging (MRIb) found no acute abnormalities and was without vasogenic edema, sequala of posterior reversible encephalopathy (PRES). After pausing tacrolimus for 4 h, the infusion was resumed and diphenhydramine injections were given as needed for a suspected dystonic reaction secondary to antiemetics. Despite cessation of antiemetics and ongoing diphenhydramine, the patient continued to have rigidity and, on day +8, he developed bilateral paresthesia of the V3 dermatomes. At this point, the diagnosis was more consistent with drug-induced parkinsonism caused by CNI neurotoxicity and tacrolimus was held. Abatacept was started for GVHD prophylaxis on day +9, also given on day +28, and then every 4 weeks until day +168 (7 total doses). MMF was continued through day +35 as planned. Day +30 bone marrow biopsy was normocellular with cleared pathogenic mutations by next-generation sequencing (NGS), chimerism >95%. Day +100 bone marrow was 40% to 50% cellular with trilineage hematopoiesis and again without pathogenic mutations, chimerism 95%. The patient had no aGHVD. He did develop biopsy proven, moderate, steroid-responsive cGVHD limited to the oropharynx at day +187 (Figure 2). At time of last follow-up, he was >1.5 years post-transplant with no active GVHD, on no immunosuppression, and overall doing well. Upon discussion with the patient, he reflects that while nervous at first to receive abatacept as part of CNI-free GVHD prophylaxis, he is glad he opted for this approach, given his “easy” post-HCT course after receiving abatacept.

## Discussion

CNI neurotoxicity was the suspected diagnosis in this case, based on the correlation with tacrolimus administration and symptom onset, paired with resolution of symptoms following tacrolimus cessation. Clinical symptoms classic for CNI neurotoxicity include headache, tremor, insomnia, and paresthesia^10^. More rare symptoms include seizures, encephalopathy, speech disorders, and cortical blindness^10,11^. PRES can occur with CNI neurotoxicity, defined by both neurological symptoms and a white matter lesion on neuroimaging^12^. CNI neurotoxicity is more common with tacrolimus; however, it also occurs with cyclosporine. CNIs limit transcription of genes necessary for production of proinflammatory cytokines, specifically IL-2, and inhibit T-lymphocyte activation. Some additional important side effects that limit CNI use include nephrotoxicity, metabolic effects (increased blood pressure, hyperglycemia, and hyperlipidemia), thrombotic microangiopathy (TMA), and rarely myelosuppression^10^. Furthermore, CNIs can be difficult to take, requiring daily compliance and frequent timed laboratory monitoring to ensure appropriate dosing to avoid both supra-therapeutic and subtherapeutic levels. Given toxicity issues and need for close monitoring, CNI use for GVHD prophylaxis can be challenging, if not impossible for many patients.

In this case, alternative immunosuppression regimens were considered, given CNI toxicity prohibiting continuation of tacrolimus. Alternative CNIs, such as cyclosporine would potentially induce neurotoxicity as well^12^. Unfortunately, to date, there are limited readily available GVHD prophylactic regimens rigorously studied and approved for use in haploidentical HCT that are CNI free^13^. Extension of MMF alone would have been insufficient immunosuppression. MMF in combination with methotrexate or sirolimus, and PTCy in combination with sirolimus, had the potential to worsen post-transplant cytopenias^3,14^. Ruxolitinib in exchange for CNI has been investigated in a small cohort of 10 patients undergoing haploidentical myeloablative HCT; however, this approach is not widely studied, is not Food and Drug Administration (FDA) approved, and also had potential to worsen cytopenias^15^. T-cell depletion *ex vivo* is another potential way to avoid CNIs, but is not ideal, given that this option must be selected upfront and thus is not useful in patients found to be CNI intolerant later in the transplant process; furthermore, this approach is not readily available at all transplant centers^13^. CTLA4-Ig blockade with abatacept was then considered and ultimately determined to be the best option for GVHD prophylaxis in this case. The FDA approved abatacept in late 2021 for GVHD prophylaxis in patients undergoing HCT from a matched unrelated donor (MUD) or 1 allele-mismatched unrelated donor (MMUD); thus, abatacept use in this case for a haploidentical HCT was off-label and offers an example of how the role of CTLA4-Ig blockade as GVHD prophylaxis, with further investigation, could potentially be expanded^8^.

**Figure 3.**
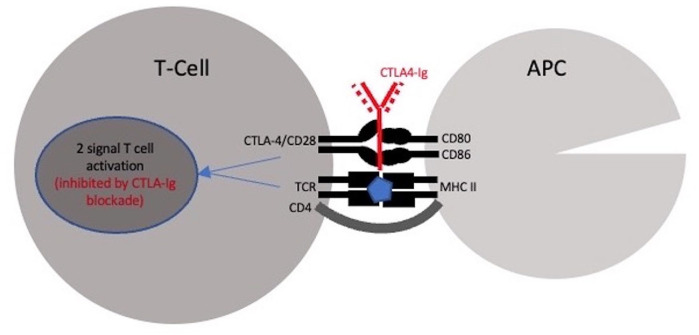
Cytotoxic T-lymphocyte-associated protein 4 (CTLA4-Ig) blockade mechanism of action at the immune synapse mediating T-cell activation. APC: antigen presenting cell; TCR: T-cell receptor; CD4: cluster of differentiation 4; CD28: cluster of differentiation 28; CD80: cluster of differentiation 80; CD86: cluster of differentiation 86; and MHC II: major histocompatibility complex II.

CTLA4-Ig blocking agents include abatacept and belatacept. These are modified antibodies that block activation of cytotoxic T-cells by binding to CD80 and CD86, preventing interaction with CTLA-4/CD28, leading ultimately to costimulation blockade (Figure 3)^16–18^. The two-signal model of T-cell activation dictates that, for T-cell activation to proceed, two signals are required: one through the antigen-specific T-cell receptor (TCR) and the second though the non-antigen-specific engagement of a costimulatory molecule by its counterpart on an antigen presenting cell. Engagement of the TCR in the absence of costimulation leads to T-cell anergy and peripheral tolerance^17,19^. Anergy is considered as the initial stage of development of regulatory T-lymphocytes and there is evidence that regulatory T-cells abolish GVHD. Extensive *in vivo* mouse studies were conducted, including initial Maurine studies, which repeatedly found that lethally irradiated mice rescued with MHC-disparate grafts had survival rates of 67% following administration of CTLA4-Ig compared with none of the mice who were not given CTLA4-Ig blockade^20^. Subsequently, a first-in-class trial was conducted in 10 MUD HCTs during which patients received four doses of abatacept and CNI/MTX, 80% of the patients had no aGVHD, while all had excellent immune reconstitution^21^. Subsequent phase 2 trials were conducted in MUD and 7/8 HLA MMUD HCT, utilizing 8 doses of abatacept in the attempt to ameliorate acute and cGVHD^22,23^. These studies also showed successful GVHD prevention with CTLA4-Ig blockade, leading to abatacept FDA approval in 2021 for select patients undergoing MUD and 7/8 HLA MMUD HCT. Our institutional experience in a randomized clinical trial has also found that omission of CNIs with a prophylactic regimen of PTCy and abatacept significantly decreased risk of acute and cGVHD, with a more favorable toxicity profile compared with traditional GVHD prophylaxis^24^. Furthermore, in haploidentical HCT, a phase 1b-2 clinical trial investigating PTCy and abatacept with CNI tacrolimus found low rates of grades 3 to 4 aGVHD (4.4%) and one year moderate-to-severe cGVHD (25.9%) rates^25^.

Although not currently approved for use in HCT, belatacept is a second-generation form of abatacept with two different amino acid substitutions that allow for high binding affinity to CD80 and CD86^18^. Belatacept is approved for immunosuppression in adult kidney transplant and is an effective option for patients unable to tolerate CNIs, despite a slightly higher risk of acute organ rejection^26^. To date, there has been one publication of two pediatric patients who successfully underwent HCT with belatacept as GVHD prophylaxis^18^. One patient was a teenager with severe aplastic anemia who was noncompliant with immunosuppression pre-transplant and then underwent matched-related donor (MRD) HCT; thus, parental belatacept was given after a short course of tacrolimus and MTX to minimize complications related to medication nonadherence to GVHD prophylaxis. The second patient was an infant with MDS who underwent MUD HCT complicated by transplant-associated TMA; thus, required alternate GVHD prophylaxis and belatacept was selected. Twenty months post-HCT, both patients were without GVHD and continued to have chimerism >90%.

Preservation of the graft-versus-leukemia (GVL) effect while preventing GVHD has been historically difficult to balance. Thus, it is paramount to consider the impact on the GVL effect when considering CNI free, CTLA4-Ig blockade-based, GVHD prophylactic regimens. GVL effect post-allogeneic HCT relies on both T cells and natural killer (NK) cells, wherein donor T cells recognize and target non-self peptides presented by self-HLA molecules and non-self HLAs, whereas donor NK cells target cells with absent or down-regulated self HLA I molecules^17,27–29^. Pre-clinical studies suggest that CTLA4-Ig blockade allows NK cell activity and thus the GVL effect remains preserved. Furthermore, our institutional experience found no significant difference in overall survival (OS), GVHD-relapse-free OS, or disease-free survival in the abatacept arm versus the CNI containing standard-of-care arm at one year post HCT^24^. Collectively this suggests that the GVL effect remains preserved with use of CTLA4-Ig blockade-based GVHD prophylactic regimens.

Currently, ongoing research is needed to better understand the long-term effects of CTLA4-Ig blockade, including the prevention of GVHD and the impact on the GVL effect. Further additional research is needed to understand the role for CTLA4-Ig blockade in HCT with higher degree of immunogenic mismatch, such as haploidentical HCT, in HCT for malignant conditions, and in adult patients. Table 1 includes a literature review of publications of haploidentical HCT utilizing CTLA4-IG blockade for GVHD prophylaxis^25,30–33^. These limited studies, predominantly in pediatric patients with nonmalignant disorders, support the safety and effectiveness of CTLA4-Ig blockade as GVHD prophylaxis in haploidentical HCT, while calling attention to the need for further investigation.

**Table 1.** Literature Review of Haploidentical Hematopoietic Cell Transplant (HCT) Utilizing Cytotoxic T-Lymphocyte-Associated Protein 4 (CTLA4-Ig) Blockade for Graft-Versus-Host-Disease (GVHD) Prophylaxis.

First author, Year	Type of study (N)	Indication for transplant (malignant/nonmalignant)	GVHD prophylaxis	Acute GVHD	Chronic GVHD	Relapse rate	Outcomes
Jaiswal et al, 2016^30^	RCT, pediatric haploidentical HCT (N = 20)	Nonmalignant (SAA)	PTCy, siroliums ± abatacept	10.5% in intervention group vs 50%	NA	NA	GVHD-free and disease-free survival at 1 year was 80% in pts treated with abatacept vs 30%.
Jaiswal et al, 2020^31^	Observational, pediatric haploidentical HCT (N = 10)	Nonmalignant (hemoglobinopathies)	Short course low dose dexamethasone, abatacept, siroliums for 6 months.	None	None	NA	No pts developed GVHD at time of median follow-up, 28 months.
Raffa et al, 2021^32^	Observational, pediatric haploidentical HCT (N = 4)	Malignant and nonmalignant (erthropoietic porphyria, primary immunodeficiency; second HCT for CML and AML)	Abatacept + MTX, MMF, and CNI (none received PTCy)	2 cases, grade 2, steroid-responsive (skin, skin and gastrointestinal)	2 cases, mild limited to skin	None	At 1.1 year, all four pts were alive with full chimerism. three were off immunosuppression.
Kharya et al, 2023^33^	RCT, pediatric haploidentical HCT (N = 79)	Nonmalignant (SAA)	PTCy, CNIs/siroliums ± abatacept	26.4%	18.9%	NA	OS and EFS were better and MVA found less GVHD in the pts treated with abatacept.
Al-Homsi et al, 2023^25^	Phase1b-2 clinical trial, haploidentical HCT (N = 46)	Malignant	PTCy, abatacept (days +5, +14, +28, and +56) and tacrolimus days +5 to +90	Grades 2 to 4: 17.4%, grades 3 to 4: 4.4%	Moderate-severe 15.9%	11.7%	Acute GVHD after haploidentical HCT was safe and effectively reduced.

The literature search was conducted from 2013 (year of first in human CTLA4-Ig blockade in human HCT) to April 2024. PubMed and Google Scholar databases were searched. Search terms included “CTLA4-Ig haploidentical HCT,” “abatacept haploidentical HCT,” and “belatacept haploidentical HCT.” RCT: randomized control trial; MVA: multivariate analysis; SAA: severe aplastic anemia; SCD: sickle cell disease; Flu: fludarabine; Cy: cyclophosphamide; ATG: antithymocyte globulin; TBI: total body irradiation; TT: thiotepa; Mel: melphalan; PTCy: post-transplant cyclophosphamide; CNI: calcineurin inhibitor; MTX: methotrexate; MMF: mycophenolate mofetil; OS: overall survival: AML: acute myeloid leukemia; CML: chronic myeloid leukemia; EFS: event-free survival; pts: patients.

## Conclusions

This case report and literature review features a number of important learning points related to current GVHD prophylaxis in haploidentical HCT. Furthermore, this case report demonstrates a potential future role for CNI-free GVHD prophylaxis utilizing CTLA4-Ig blockade in haploidentical HCT that warrants future investigation.
